# USP7 Is a Master Regulator of Genome Stability

**DOI:** 10.3389/fcell.2020.00717

**Published:** 2020-08-05

**Authors:** Gabrielle J. Valles, Irina Bezsonova, Roger Woodgate, Nicholas W. Ashton

**Affiliations:** ^1^Department of Molecular Biology and Biophysics, UConn Health, Farmington, CT, United States; ^2^Laboratory of Genomic Integrity, National Institute of Child Health and Human Development, National Institutes of Health, Bethesda, MD, United States

**Keywords:** ubiquitin, mutagenesis, genomic integrity, replication, DNA repair, tumor suppressor protein p53, cancer

## Abstract

Genetic alterations, including DNA mutations and chromosomal abnormalities, are primary drivers of tumor formation and cancer progression. These alterations can endow cells with a selective growth advantage, enabling cancers to evade cell death, proliferation limits, and immune checkpoints, to metastasize throughout the body. Genetic alterations occur due to failures of the genome stability pathways. In many cancers, the rate of alteration is further accelerated by the deregulation of these processes. The deubiquitinating enzyme ubiquitin specific protease 7 (USP7) has recently emerged as a key regulator of ubiquitination in the genome stability pathways. USP7 is also deregulated in many cancer types, where deviances in USP7 protein levels are correlated with cancer progression. In this work, we review the increasingly evident role of USP7 in maintaining genome stability, the links between USP7 deregulation and cancer progression, as well as the rationale of targeting USP7 in cancer therapy.

## Introduction

Maintaining the genomic integrity of cells is vital, as alterations to the genetic code can result in deregulation of cellular function, malignant transformation or cell death. This can lead to a variety of disorders including neurological degeneration, premature aging, developmental defects and cancer (Khanna and Jackson, 2001). To prevent genetic alterations, cells employ a range of genome stability pathways, which allow for the accurate metabolism of the DNA, as well as for any DNA errors or damage to be rapidly repaired (Hoeijmakers, 2001). Post-translational modifications play an essential role in the signaling, activation and coordination of the genome stability pathways (Polo and Jackson, 2011). The reversable ubiquitination of proteins is one such essential modification that regulates numerous key factors involved in genome stability. Known as the “ubiquitin code”, this versatile pattern of mono- or polyubiquitin moieties dynamically regulates the activity, interactions, localization and stability of substrate proteins (Komander and Rape, 2012). For example, ubiquitination promotes initiation of DNA replication (Hernandez-Carralero et al., 2018), regulates all DNA damage tolerance and repair pathways and activates cell cycle regulatory components (Mocciaro and Rape, 2012). Dynamic ubiquitination is therefore essential to prevent genome instability.

Ubiquitination is mediated by a cascade of E1, E2 and E3 ubiquitin enzymes, which covalently attach the 8.5 kDa ubiquitin protein onto a substrate molecule, while deubiquitinating enzymes (DUBs) can edit or remove ubiquitin modifications (Pickart and Eddins, 2004). The human genome encodes close to 100 DUBs, which can be structurally grouped into seven distinct protein families: ubiquitin specific proteases (USPs), ovarian tumor proteases (OUTs), JAB1/MPN/MOV24 metalloproteases (JAMMs), ubiquitin c-terminal hydrolases (UCHs), Macho-Joseph disease protein domain proteases (MHDs), motif interacting with ubiquitin (MIU)-containing novel deubiquitinases (MINDYs) and ZUP1 (Ruan et al., 2020). Of these, the ubiquitin specific proteases (USPs) represent the largest DUB family, with 57 members (Komander et al., 2009). Many USPs have been found to function within the genome stability pathways (Jacq et al., 2013). USP1, for instance, regulates the monoubiquitination of FANCD2 (Oestergaard et al., 2007; Liang et al., 2019) and PCNA (Huang et al., 2006), thereby influencing the pathways of interstrand crosslink repair and translesion synthesis, respectively. By contrast, USP47 deubiquitinates and stabilizes polyubiquitinated DNA polymerase β, ensuring availability of the latter to participate in base excision repair (Parsons et al., 2011). While many USPs play essential roles in ensuring genome stability, they are often dysregulated in cancers where they promote tumorigenesis (Young et al., 2019). The prospect of targeting USPs has thus been increasingly considered as an attractive approach for cancer therapies (Yuan et al., 2018).

Ubiquitin specific protease 7 (USP7), also known as Herpesvirus associated protease (HAUSP), is an ∼128 kDa cysteine protease and member of the USP DUB family. USP7 was first identified as an interacting partner of herpes simplex virus type 1 (HSV-1) regulatory protein infected cell polypeptide 0 (ICP0) (Everett et al., 1997). Since its discovery, mounting evidence has demonstrated the extensive roles and interaction network of USP7 in several cellular pathways. This includes its significant role in genome stability, where USP7 regulates the well-characterized p53/Mdm2 signaling axis (Li et al., 2004), along with many other factors that will be addressed in detail in this review. USP7 is an essential enzyme; its homozygous knockout in mice results is early embryonic lethality (Kon et al., 2010) and no human individuals have been identified who are USP7 homozygous-null (Fountain et al., 2019). Heterozygous loss-of-function USP7 mutations have, however, been identified in 23 individuals, all of whom have experienced neurodevelopmental disorders – characterized by developmental delay/intellectual disability (DD/ID), speech delay, behavioral anomalies and autism spectrum disorder – as well as physical characteristics that include dysmorphic facial features (Hao et al., 2015; Fountain et al., 2019). While diseases caused by pathogenic somatic mutations of USP7 are rare, the aberrant expression of USP7 is much more frequent, resulting in deregulation of numerous pathways and contributing to disease states that include non-small cell lung cancer (Masuya et al., 2006) and prostate cancer (Song et al., 2008a). In this review, we will discuss the many roles USP7 plays in maintaining genome stability and its links with cancer.

## Overview of USP7 Structure

**FIGURE 1:**
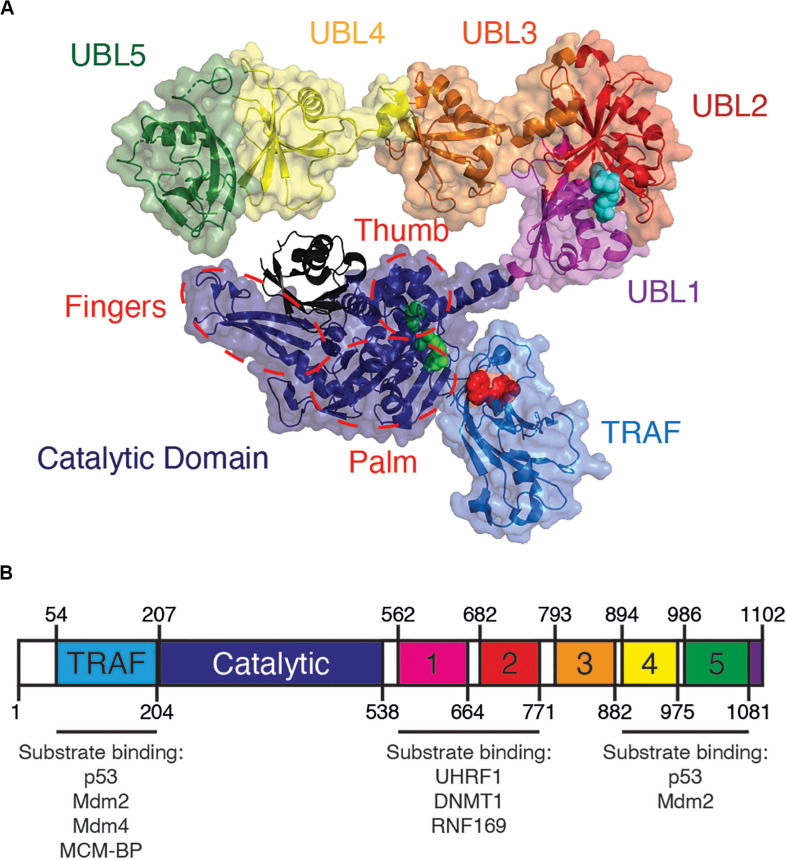
Overview of USP7 domain structure. **(A)** USP7 model based on superposition of PDBs 2f1z, 5fwi and 4z97, with ubiquitin (black) (PDB ID:1nbf). Red spheres on the TRAF-like domain indicate the residues D164 and W165 responsible for substrate binding. Green spheres on catalytic domain represent catalytic triad active site residues C223, H464, and D481. Blue spheres on UBL2 indicate substrate-binding residues D758, E759, and D764. Red dashed lines indicate the characteristic papain-like fold “thumb, palm, fingers” sub-domain architecture of the USP7 catalytic domain. **(B)** Schematic of USP7 indicating the amino acid location of each domain, as well as of each substrate-binding region. Lists below the schematic indicate protein substrates known to bind each substrate-binding site.

All USPs share a conserved catalytic core, while their unique substrate specificity is determined by a variety of accessory substrate-binding domains (Nijman et al., 2005). From its N- to C-terminus, USP7 contains a tumor necrosis factor receptor associated factor-like (TRAF-like) domain, a catalytic domain and five ubiquitin-like (UBL) domains (Figure 1). Although a full-length structure of USP7 is yet to be determined, structural information is known for each individual USP7 domain (Hu et al., 2001; Saridakis et al., 2005; Faesen et al., 2011a).

### Substrate Recognition by TRAF-Like Domain

At its very N-terminus, USP7 contains an intrinsically disordered ∼50 amino acid sequence with a polyglutamine region, followed by its TRAF-like domain (residues 52–205). The USP7 TRAF-like domain has been extensively characterized as one of two major substrate-binding sites within USP7. Of the USP family of enzymes, USP7 is the sole member with a TRAF-like domain. This endows USP7 with unique substrate recognition and specificity that sets it apart from other USPs (Nijman et al., 2005).

USP7 is primarily nuclear and the N-terminus of the enzyme is largely responsible for its nuclear localization. Thus, truncation of amino acids 1–207 abolishes its ability to enter the nucleus. As this region of the protein does not contain a nuclear localization signal, it has been speculated that binding to USP7 substrates – which are primarily nuclear – may mediate translocation of USP7 into the nucleus (Zapata et al., 2001; Fernandez-Montalvan et al., 2007).

The majority of known USP7 substrate interactions occur within the TRAF-like domain. This wide range of substrates includes several factors that are involved in genome stability. The interactions between the USP7 TRAF-like domain and its substrates has been extensively characterized structurally. The TRAF-like domain assumes an eight stranded antiparallel β- sandwich fold, with residues D164 and W165 forming essential contacts with P/A/ExxS motifs shared by all TRAF-like domain-binding substrates. These substrates include the tumor suppressor p53 (Hu et al., 2006; Sheng et al., 2006), the Mdm2 E3 ubiquitin ligase (Hu et al., 2006; Sheng et al., 2006; Sarkari et al., 2010), the Mdm2 homolog Mdm4 (Sarkari et al., 2010) and mini-chromosome maintenance binding-protein MCM-BP (Jagannathan et al., 2014; Figure 1 and Table 1).

**TABLE 1:** List of USP7 substrates involved in genome stability and maintenance with available structural information.

**USP7 substrate**	**USP7 interaction domain**	**K_d_ (μM)**	**PDB ID**
p53	TRAF	18 ± 1.5	2foo, 2foj, 2f1x
Mdm2	TRAF	8 ± 0.3	2fop, 2f1y, 3mqs
Mdm4	TRAF	N/A	3mqr
MCM-BP	TRAF	N/A	4kg9
UHRF1	UBL1-2	1.5	5c6d
DNMT1	UBL 1-2	0.6 ± 0.05	4yoc
RNF169	UBL 1-2	1.5 ± 1.1	5gg4

As all USP7 TRAF-like substrate interactions occur on an identical interface, these interactions occur in a mutually exclusive manner. This regulation of TRAF-like-mediated interactions is important for fine-tuning ubiquitination of USP7 substrates (as is described in the “***p53-Dependent Transcription”*** section below).

### USP7 Catalytic Domain

Following the TRAF-like domain and central to USP7 is its catalytic domain (residues 207–560). The catalytic domains of USPs contain a highly conserved Cysteine-Histidine box, which in USP7 is formed by residues C223, H464 and D481 (Hu et al., 2001).

Despite differences in size, the catalytic domains of all USPs possess a characteristic papain-like fold with a “thumb, palm, fingers” sub-domain architecture (Reyes-Turcu et al., 2009) (Figure 1). The fingers “hold” ubiquitin and is the primary interaction interface between the enzyme and ubiquitin. The active site catalytic triad resides in a cleft between the “palm” and “thumb” of the enzyme. The C-terminal tail of ubiquitin conjugated to a lysine residue of the substrate enters this cleft, allowing for hydrolysis of the isopeptide bond between ubiquitin and the substrate (Hu et al., 2001; Pozhidaeva et al., 2017). Once cleaved, the low affinity between free ubiquitin and the catalytic domain results in subsequent release of the product (Faesen et al., 2011b; Pozhidaeva et al., 2017; Kim et al., 2019). Accordingly, mutations that enhance ubiquitin affinity to USPs result in inhibition of the enzyme (Morrow et al., 2018)

Crystal structures of the USP7 catalytic domain apo-enzyme (PDB ID: 1nb8) and the USP7 catalytic domain in complex with ubiquitin-aldehyde (PBD ID: 1nbf), reveal that for the most part, ubiquitin-binding does not result in broad conformational changes to the catalytic domain. However, major structural deviations do occur for the active site catalytic triad residues (C223, H464, D481), which undergo drastic conformational movement upon ubiquitin binding. In the apo-enzyme or “inactive” catalytic domain structure, active site residues are too far apart to perform enzymatic functions, while these same residues move towards each other in the “active” ubiquitin-bound structure. The “switching loop,” a loop that is proximal to the active site, undergoes even larger rearrangement and becomes more structured upon ubiquitin-binding (Hu et al., 2001; Faesen et al., 2011a). The existence of these two distinct conformations assumed by the USP7 catalytic domain may be a mechanism employed by the enzyme to regulate its activity (Hu et al., 2001; Ozen et al., 2018).

### C-Terminal UBL Domains Regulate Activation and Specificity of USP7

A long and potentially flexible α-helix connects the USP7 catalytic domain to its 64 kDa C-terminal ubiquitin-like domains (UBL 1–5) (Kim et al., 2016). Of the USPs, USP7 has the greatest number of UBLs (Komander et al., 2009). Despite low sequence homology to each other and to ubiquitin, these UBLs all structurally resemble the classic β-grasp ubiquitin fold (Faesen et al., 2011a, 2012).

USP7 UBLs function as di-UBL units in a 2-1-2 domain architecture: UBL 1-2, UBL 3, and UBL 4-5 (Kim and Sixma, 2017). These units are separated by flexible linkers, which allow for the C-terminus to assume an extended or compact conformation. This conformational flexibility and allosteric regulation is required for enzymatic activity (Faesen et al., 2011a, 2012; Pfoh et al., 2015; Kim et al., 2016; Rouge et al., 2016). While the catalytic domain is essential for enzymatic activity of USP7, the isolated catalytic domain has poor catalytic activity compared to the full-length enzyme (Faesen et al., 2011a). Interestingly, efficient enzymatic activity of USP7 requires its UBL 4-5 domains (amino acids 894–1102). Studies have shown that following ubiquitin binding to the catalytic domain, the USP7 C-terminal domains assume a conformation that allows its disordered tail (residues 1084–1102) to bind to the “switching loop.” This conformational switch promotes an “active” state, increasing USP7 enzymatic activity. Without UBL 4–5, the catalytic activity of USP7 is severely compromised. Therefore, a functional full-length USP7 is required for its proper activity (Faesen et al., 2011a; Rouge et al., 2016; Kim et al., 2019).

### UBL Domains Also Function as Additional Platform for Substrate-Binding

Outside of the TRAF-like domain, the UBL 1–2 domains contain the second major substrate recognition site for USP7 substrates. These interactions largely occur through an acidic patch on the interface of UBL 2 (Figure 1). This acidic interface interacts with a highly basic motif (R/KxKxxxK) within USP7 substrates that include UHRF1 (Zhang et al., 2015), DNMT1 (Cheng et al., 2015) and RNF169 (An et al., 2017; Figure 1 and Table 1).

UBL 4–5 has also been speculated to contain a secondary binding site outside of the TRAF-like domain for binding to p53 and Mdm2, although further validation of this site is still needed (Ma et al., 2010; Kim et al., 2019).

## USP7 Cleaves Mono- and Polyubiquitin Chains From Substrate Proteins

The “ubiquitin code,” or pattern of ubiquitin modifications that occurs on a substrate, serves to regulate tagged substrates (Komander and Rape, 2012). Proteins may be monoubiquitinated, where a single ubiquitin moiety is covalently attached to a lysine residue of a substrate, or polyubiquitinated, where the initial ubiquitin group is further ubiquitinated. Successive ubiquitination can occur at one of the seven lysine residues within ubiquitin – K6, K11, K27, K29, K33, K48, and K63 – to form polyubiquitin chains of specific linkage types. Besides lysine residues, the N-terminal methionine of ubiquitin can also be ubiquitinated (Spit et al., 2019). USP7 specifically cleaves ubiquitin and is unable to cleave other ubiquitin-like modifiers including SUMO and Nedd8 (Fernandez-Montalvan et al., 2007).

The pattern of ubiquitin linkage on a substrate can have several effects, including targeting the substrate for proteasomal or lysosomal degradation, influencing substrate localization, regulating protein-protein interactions, and altering substrate activity (Komander and Rape, 2012). To date, the most common and well-characterized ubiquitin modifications include K11 and K48 polyubiquitin chains, which are best known for targeting substrates for proteasomal degradation and K63 polyubiquitin chains, which can signal proteins for lysosomal targeting and NF-KB activation (Komander and Rape, 2012). K63 polyubiquitination, as well as monoubiquitination, can also generate binding-platforms to mediate specific protein-protein interactions. The USP7 substrate PCNA, for instance, can be modified by both mono- and K63 polyubiquitination, which mediates interactions with translesion DNA polymerases (Bienko et al., 2005) and ZRANB3 (Vujanovic et al., 2017), respectively (discussed further in the ***DNA Damage Bypass*** section below).

Although USP7 substrate-specificity has been extensively characterized, its ubiquitin chain specificity for its specific substrates is still being elucidated. Like other USPs, USP7 is unable to cleave methionine-linked linear ubiquitin chains (Faesen et al., 2011a). USP7 can, however, cleave K6, K11, K33, K48, and K63-linked modifications and does so with similar efficiencies (Faesen et al., 2011a; Schaefer and Morgan, 2011; Kategaya et al., 2017; Masuda et al., 2019). As a means of regulating substrate-specificity, USP7 is inefficient in recycling and converting free-floating polyubiquitin chains to monoubiquitin if the chain is not attached to a substrate (Schaefer and Morgan, 2011; Masuda et al., 2019). USP7 can, however, cleave ubiquitin chains that are attached to the small ubiquitin-like modifier, SUMO, regardless of whether SUMO is itself attached to another substrate or otherwise free-floating (Lecona et al., 2016).

**FIGURE 2:**
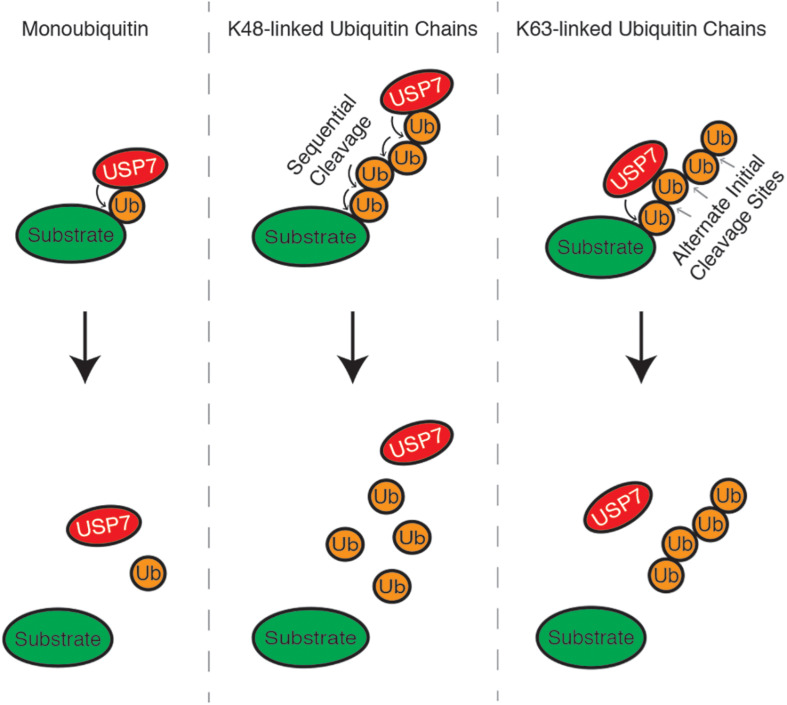
USP7 can cleave monoubiquitin moieties, as well as K48 and K63-linked polyubiquitin chains, from substrate proteins. K48 polyubiquitin chains are cleaved from the distal end of the chain, working inwards, while linkages between K63 polyubiquitin chains can be cleaved indiscriminately.

K63 and K48 polyubiquitin chains are the best-characterized ubiquitin linkages cleaved by USP7. USP7 indiscriminately cleaves K63 polyubiquitin linkages throughout the chain. This includes cleavage between ubiquitin and the substrate (base cleavage), between two ubiquitin molecules within the chain (endo-cleavage) or at the distal end of the chain, working inwards (exo-cleavage) (Figure 2). One study suggests that K48-polyubiquitin linkages are not favored by USP7 and that the enzyme prefers ubiquitin modifications with a free K48 side chain. Because of this, USP7 preferentially exo-cleaves K48 polyubiquitin linkages, rather than endo-or base cleavage. This preference may act as an intrinsic negative regulator of USP7, helping to ensure that proteins destined for proteasomal degradation are in fact degraded (Kategaya et al., 2017). USP7 activity is also influenced by the length of the polyubiquitin chain, as long polyubiquitin chains (>4 ubiquitin) seem to be “resistant” to cleavage compared to shorter chains. This chain specificity is speculated to be an additional mechanism regulating USP7 activity, by serving as a threshold for proteasome-targeting polyubiquitination (Schaefer and Morgan, 2011; Kategaya et al., 2017).

Despite the limitations above, stabilizing proteins by removal of proteasome-targeting K48 polyubiquitin chains is a common mechanism by which USP7 regulates many of its characterized substrates. Remarkably, these USP7 substrates include a high number of E3 ubiquitin ligases and their target substrates (Kim and Sixma, 2017). This results in an interesting dichotomy, where USP7 can deubiquitinate and stabilize: 1) the E3 ubiquitin ligase, allowing it to mediate K48 ubiquitination of its substrates; or 2) the substrate targeted by the E3 ubiquitin ligase. Most intriguing is the frequent observation for this pattern to occur as part of a negative feedback system, where USP7 contributes to both stabilizing and degrading both substrates. This arrangement allows USP7 to fine-tune substrate protein levels and dynamically regulate its target pathways.

## USP7 Is a Master Regulator of Genomic Integrity Pathways

In this section, we will discuss the intricate manner through which USP7 regulates the genome stability pathways. We will firstly discuss the roles of USP7 in maintaining genome stability during normal cellular metabolism, by regulating the cell cycle, replication, mitosis and telomere stability. We will then review roles of USP7 following the induction of DNA damage, in regulating p53-dependent transcription and the pathways of nucleotide excision repair, double-strand DNA break repair and DNA damage bypass. Finally, we will consider roles of USP7 in the ‘last resort’ pathway of apoptosis.

### Cell Cycle Regulation

For a proliferating cell to give rise to identical daughter cells, genomic DNA must be duplicated and equally distributed into two new nuclei. These events occur in the S and M phases of the cell cycle; DNA is replicated during S phase, and then separated into distinct nuclei via mitosis in M phase. Towards the end of M phase, these two nuclei are partitioned into separate daughter cells in the process of cytokinesis. Interspersing S phase and M phase are two growth phases – G_1_ and G_2_ – during which the cell synthesizes new mRNA and protein for the subsequent phase. Progression through the cell cycle – G_1_, S, G_2_, M – is tightly regulated by proteins referred to as cyclins, which activate cyclin-dependent kinases (CDKs) to phosphorylate key proteins that initiate each cell cycle phase (Malumbres and Barbacid, 2009).

**FIGURE 3:**
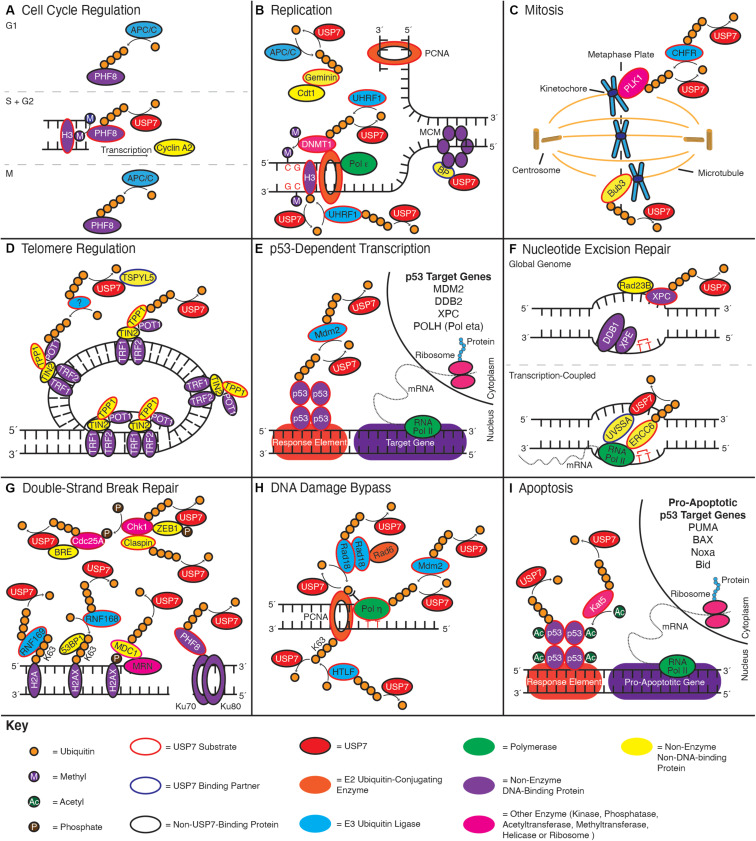
USP7 functions in numerous genome stability pathways. Schematics indicate roles of USP7 in: **(A)** cell cycle regulation, **(B)** replication, **(C)** mitosis, **(D)** telomere regulation, **(E)** p53-dependent transcription, **(F)** nucleotide excision repair, **(G)** double-strand DNA break repair, **(H)** DNA damage bypass, and **(I)** apoptosis. Curved arrows towards a substrate indicate ubiquitination events, while curved arrows away from a substrate indicate deubiquitination; enzymes adjacent to the arrow catalyze the reaction. Color coding throughout the figure is indicated by the Key at the bottom of the figure.

Numerous reports have demonstrated that the inhibition or depletion of USP7 in cells causes defects in cell cycle progression and a resulting decrease in cell proliferation (Reverdy et al., 2012; Giovinazzi et al., 2013; Jagannathan et al., 2014; Yi et al., 2016). While these phenotypes are multi-factorial – resulting from disruptions to DNA replication, mitosis, and DNA repair – mounting evidence suggests they are also the result of disrupted cell cycle regulation. This includes recent findings regarding a role of USP7 in regulating the expression of cyclin A2, one of the major regulators of S phase initiation and the subsequent transition to G2 (Figure 3A; Wang et al., 2016).

USP7 regulates cyclin A2 indirectly, by stabilizing the PHF8 demethylase (Wang et al., 2016). PHF8 activates transcription start sites by demethylating the histone substrates H3K9me^1/2^, H3K27me^2^, and H4K20me^1^. PHF8 also has an integral role in the upregulation of gene targets involved in cell cycle progression, including cyclin A2 (Liu et al., 2010). Protein levels of PHF8 are themselves cell cyclically regulated, with levels peaking in G2 due to USP7-mediated deubiquitination, before being degraded in M and G1 due to polyubiquitination by the anaphase-promoting complex (APC/C) (Lim et al., 2013; Wang et al., 2016).

Another means through which USP7 regulates cell cycle progression is via stabilization of the Ki-67 antigen (Zhang et al., 2016). Ki-67 is a nuclear protein often used as biomarker of cell proliferation in immunohistochemical analysis of malignant tissues (Sales Gil and Vagnarelli, 2018). This is due to the near-exclusive expression of Ki-67 in the active cell cycle phases (G_1_, S, G_2_, M), as opposed to in the quiescent ‘G_0_’ state of non-dividing cells. Although Ki-67 expression has been used as a proliferation marker for many decades, roles for this protein remain poorly described. Recent findings, however, suggest that Ki-67 contributes to cell cycle regulation, heterochromatin maintenance and mitosis (Sun and Kaufman, 2018).

### Replication

In addition to broad cell cycle regulation, USP7 also has more direct roles in regulating DNA replication (Figure 3B). One of the earliest steps in this process is the so-called ‘licensing’ of origins of replication (Bell, 2017). This process occurs in late M phase/G1 and involves recruiting mini-chromosome maintenance 2–7 (MCM 2–7) replicative helicases to thousands of potential replication start sites, ready for use during S phase. Replication licensing is mediated by the licensing factors Cdc6 and Cdt1, which help to load the MCM proteins onto DNA (Evrin et al., 2009; Remus et al., 2009). To ensure that origins of replication can only be fired once per cell cycle, licensing is strictly inhibited once the cell enters S phase. This is essential to prevent DNA being replicated more than once per cell cycle, an undesirable outcome which can cause genome instability and tumorigenesis as a result of gene amplification (Truong and Wu, 2011).

USP7 has a role in preventing DNA re-replication by stabilizing Geminin, a negative regulator of replication (Hernández-Pérez et al., 2017). Geminin interacts directly with Cdt1, preventing Cdt1from loading MCM proteins onto origins of replication (Wohlschlegel et al., 2000). Analogous to the regulation of PHF8, Geminin protein levels are cyclically regulated to increase in S and G2 due to USP7-mediated deubiquitination, prior to degradation in M phase and G1 as a result of APC/C-mediated polyubiquitination (McGarry and Kirschner, 1998). By deubiquitinating Geminin in S and G2, USP7 helps to ensure its availability to suppress aberrant origin licensing (Hernández-Pérez et al., 2017).

The firing of replication origins occurs once the MCM2-7 proteins associate with Cdc45 and the GINS protein complex, to form an active replicative helicase (Gambus et al., 2006; Moyer et al., 2006; Ilves et al., 2010). This complex then begins the process of unwinding the DNA, allowing the recruitment of ‘replisome’ proteins, while exposing single-stranded DNA for use as a template for new DNA synthesis by replicative DNA polymerases (Leman and Noguchi, 2013). A recent study has indicated that USP7 associates at sites of replication and is important for origin firing and replication fork progression (Lecona et al., 2016). Here, USP7 deubiquitinates replication fork proteins, with an apparent preference for substrates that are also SUMOylated. This role allows USP7 to maintain a high local ratio of SUMOylated to ubiquitinated proteins, a distinguishing feature of replication fork-adjacent chromatin (Lopez-Contreras et al., 2013). An explanation for this role is that by deubiquitinating replication proteins, USP7 is able to prevent their recognition by the valosin-containing protein (VCP) [also known as the p97 segregase (Lecona et al., 2016)]. VCP recognizes and extracts chromatin-bound ubiquitinated proteins, allowing for their proteasomal degradation (Dantuma and Hoppe, 2012). By deubiquitinating SUMOylated proteins at the replication fork, USP7 may therefore help to maintain protein levels required for new DNA synthesis. A precise catalog of SUMOylated proteins targeted by USP7, will, however, require further exploration.

USP7 also contributes to maintaining the methylation status of newly synthesized DNA through multi-level regulation of DNA (cytosine-5)-methyltransferase 1 (DNMT1) (Felle et al., 2011; Qin et al., 2011). DNMT1 catalyzes the methylation of cytosine bases at cytosine-phosphate-guanine (CpG) dinucleoside sites and as such is an important mediator of epigenetic gene silencing (Jones et al., 1998; Dhe-Paganon et al., 2011). Unlike related DNA methyltransferases of the DNMT3 family, which have a higher affinity for non-methylated DNA, DNMT1 instead preferences the methylation of hemi-methylated CpG sites (Hermann et al., 2004). This preference allows DNMT1 to specifically modify newly synthesized DNA by copying the methylation pattern of the template strand. Although an association with the replication fork is not strictly necessary for the post-replicative activity of DNMT1, DNMT1 does interact directly with the PCNA sliding clamp. This interaction is thought to increase the catalytic efficiency of DNMT1 (Chuang et al., 1997; Schermelleh et al., 2007; Spada et al., 2007).

USP7 has been found to interact directly with DNMT1 and was initially suggested to stabilize the latter by opposing its polyubiquitination by the E3 ubiquitin ligase UHRF1 (Felle et al., 2011; Qin et al., 2011). Acetylation of DNMT1 by Kat5 (also known as Tip60) at the end of S phase was further proposed to disrupt the USP7-DNMT1 interaction and target the latter for degradation (Du et al., 2010; Cheng et al., 2015). Some doubt has, however, recently arisen as to the extent to which USP7 contributes to DNMT1 stability (Yarychkivska et al., 2018). Indeed, in this report, DNMT1 protein levels were found to be unaffected by USP7-depletion or to decrease at the end of S phase. Reconciling these findings will require further study.

While the role of USP7 in regulating DNMT1 protein levels remains unclear, numerous reports have indicated that USP7 also regulates DNMT1 chromatin localization via indirect means. This includes USP7-mediated stabilization of UHRF1 (Jenkins et al., 2005; Felle et al., 2011). In addition to its role in polyubiquitinating DNMT1, UHRF1 is also a critical factor in localizing DNMT1 to chromatin (Bostick et al., 2007; Achour et al., 2008; Bronner et al., 2019). UHRFI has been suggested to guide DNMT1 towards hemi-methylated DNA, by cooperative binding to both hemi-methylated DNA and lysine 9 methylated histone H3 (H3K9me^3^) (Liu et al., 2013). USP7 has been suggested to have an additional role in this step, where its binding to UHRF1 seems to promote a conformational change in UHRF1, allowing for efficient H3K9me^3^-binding (Gao et al., 2018). These rearrangements presumably contribute to the subsequent channeling of DNMT1 to these histone marks. In addition, UHRF1 seems to promote DNMT1 chromatin binding by monoubiquitinating histone H3 at three different lysine residues, K14, K18, and K23, which generates a platform for DNMT1-binding (Nishiyama et al., 2013; Harrison et al., 2016; Ishiyama et al., 2017). Interesting, USP7 seems to play a role in negatively regulating this interaction by deubiquitinating H3 (Yamaguchi et al., 2017); this may allow USP7 to fine-tune DNMT1 recruitment.

USP7 also has a likely role in the final stages of replication, in the unloading of MCM complexes. This was first suggested by the delayed progression of USP7-depleted cells through late S and G2 of the cell cycle, which corresponds with a defect in the dissociation of MCM proteins from chromatin (Jagannathan et al., 2014). Furthermore, USP7 was found to interact with MCM-binding protein (MCM-BP) – a protein that mediates MCM disassembly and dissociation from DNA following the completion of replication (Nishiyama et al., 2011; Nguyen et al., 2012). Although a precise mechanism remains unclear, these observations suggest USP7 has a direct role in the disassembly of replication forks, presumably involving an interaction with MCM-BP.

### Mitosis

While the accurate replication of DNA is essential to prevent the accumulation of DNA mutations, the precise division of this DNA during mitosis is equally important for the generation of genetically identical daughter cells. Indeed, failures during this process can result in aneuploidy – the production of daughter cells with too many or too few chromosomes – as well as other chromosomal rearrangements (Levine and Holland, 2018). The consequence of these errors is highlighted by the structural alterations and copy number changes of chromosomes found in the vast majority of tumors (Duijf et al., 2013). A number of studies have suggested that USP7 functions in the regulation of mitosis (Oh et al., 2007; Giovinazzi et al., 2013, 2014; Peng et al., 2019; Figure 3C). Here, cells depleted of USP7 exhibit increases in chromosomal abnormalities and aneuploidy, as well as an accumulation of micronuclei (Giovinazzi et al., 2013, 2014).

Mitosis occurs as a series of five distinct phases: prophase, prometaphase, metaphase, anaphase, telophase (McIntosh, 2016). In prophase, the duplicated and entangled chromosomes are condensed into two distinct sister chromatids, joined together by a centromere (Belmont, 2006). Disk-shaped protein structures called kinetochores then assemble on the centromere in prometaphase, which are bound by microtubules originating from centrosomes found at either end of the cell (Magidson et al., 2011). The chromosomal centromeres are then aligned at equal distances from the centrosomes in metaphase, along the so-call metaphase plate (Jaqaman et al., 2010). In anaphase, the sister chromatids are then separated and move to opposite ends of the cell, prior to formation of distinct nuclear membranes around each identical set of chromatids in telophase (McIntosh, 2016).

One of the major ways USP7 contributes to mitosis, is by regulating the stability of polo-like kinase 1 (PLK1) (Giovinazzi et al., 2013; Peng et al., 2019). PLK1 is one of the foremost M phase kinases, which functions in almost every stage of mitosis, including progression through the G2/M boundary, the alignment of chromatids on the metaphase plate and sister chromatid segregation (Combes et al., 2017). USP7 both positively and negatively influences PLK1 proteins levels by deubiquitinating and stabilizing both PLK1 and its E3 ubiquitin ligase, CHFR (Oh et al., 2007; Peng et al., 2019). CHFR triggers the degradation of PLK1 in response to mitotic stress, which prevents activation of mitosis-promoting factor and progression into M phase (Kang et al., 2002). By stabilizing both PLK1 and CHFR, USP7 thereby helps to regulate mitotic entry; indeed, cells depleted of USP7 exhibit G2/M cell cycle arrest and delayed mitotic progression (Giovinazzi et al., 2013; Peng et al., 2019). How the cell balances these opposing functions of USP7 will, however, require further study.

In addition to mitotic entry, USP7-mediated stabilization of PLK1 also contributes to the PLK1 regulation of chromatid alignment and segregation. Indeed, cells depleted of PLK1 or USP7 exhibit increased rates of chromosomal misalignment and segregation during metaphase and anaphase, respectively (Peng et al., 2019). PLK1 exerts these functions by localizing to the chromatid kinetochores in prometaphase, where it promotes microtubule attachments (Liu et al., 2012).

Aside from PLK1-mediated regulation, USP7 also influences proper chromatid alignment and segregation by deubiquitinating Bub3 during M phase (Giovinazzi et al., 2014). Bub3 is a key component of the Spindle Assembly Checkpoint (SAC), where it functions with other members of the Mitotic Checkpoint Complex (MCC) (Musacchio, 2015). The SAC regulates the metaphase-to-anaphase transition, by ensuring that all sister chromatids are aligned on the metaphase plate and properly connected to centrosomal microtubules – a process regulated by PLK1 (Combes et al., 2017) – prior to chromatid segregation during anaphase. The SAC delays this transition by inhibiting the anaphase-promoting complex (APC/C) and preventing it from degrading cyclin B and securin (Musacchio, 2015). Securin, in turn, inhibits the protease separase, which is responsible for cleaving the cohesin rings that hold sister chromatids together to initiate anaphase (Ross and Cohen-Fix, 2002). By stabilizing both PLK1 and Bub3, USP7 therefore regulates chromosomal segregation in two ways: 1) by regulating the proper attachment of microtubules to the kinetochores, and 2) by ensuring a proper checkpoint response can be initiated, in the event of any errors.

### Telomere Maintenance

Another means by which USP7 influences genome stability, is through the maintenance of telomere ends (Figure 3D; Zemp and Lingner, 2014; Episkopou et al., 2019). Telomeres are protein-nucleotide structures at the termini of each eukaryotic chromosome, which in humans contain 10 to 15 kb of a repeating hexanucleotide sequence (Kong et al., 2013). While the vast majority of the telomeric DNA is double-stranded, the termini of each telomere is characterized by a 3’ overhang of 50–300 bp of single-stranded DNA (ssDNA). These overhangs result from the so called “end-replication problem,” where the cell is unable to replicate the 3’ termini of chromosomes due to the strict requirement of a primed template from which replication can proceed (Harley et al., 1990). These overhangs represent a challenge for the cell, as exposed ssDNA is vulnerable to both chemical and enzymatic degradation. Furthermore, as telomere ends resemble broken chromosomes, they must be protected to prevent triggering a double-strand DNA break response and the fusion of adjacent chromosomes (O’Sullivan and Karlseder, 2010).

One of the ways cells sequester telomere overhangs is through the formation of so called “t-loops”. These structures form when the telomeric ssDNA overhang invades an upstream region of the telomere duplex and stably binds via complementarity of the hexanucleotide sequence (Griffith et al., 1999). Although this protects the telomere overhang, it does so at the expense of displacing a section of ssDNA from the upstream DNA duplex. This displaced section is, however, rapidly bound by proteins of the shelterin complex – comprised of TRF1, TRF2, POT1, TPP1, TIN2, and Rap1 – which bind both directly to the ssDNA section, as well as the adjacent dsDNA (de Lange, 2005). In germ cells and the vast majority of cancer cells, the shelterin complex also contributes to regulating telomere lengthening, either via telomerase-mediated DNA synthesis (active in 85 – 90% of cancers) or recombination-based alternative lengthening of telomeres (ALT; active in 10 – 15% of cancers) (Cesare and Reddel, 2010).

USP7 contributes to telomere maintenance by interacting with and deubiquitinating the shelterin component, TPP1 (Zemp and Lingner, 2014). TPP1 binds to telomeric ssDNA as a heterodimer with POT1, which helps to protect the telomere from degradation. In addition, TPP1 interacts directly with telomerase and is a processivity factor for telomerase-mediated telomere lengthening (Wang et al., 2007; Xu et al., 2019). Unlike murine TPP1, whose ubiquitination promotes telomere localization (Rai et al., 2011), the ubiquitination of human TPP1 signals the protein for proteasomal degradation. USP7, however, stabilizes human TPP1, likely in conjunction with other DUBs (Zemp and Lingner, 2014). Although a precise functional outcome of USP7-mediated TPP1 stabilization remains unclear, by limiting TPP1 degradation, USP7 presumably contributes to the integrity of telomere cap as well as regulation of telomerase-mediated end lengthening.

USP7 localizes to telomere ends in both telomerase^+^ and ALT^+^ human cell lines, however, its roles at either seem to differ. Indeed, while USP7 has an apparent protective role in telomerase^+^ cells – via TPP1 stabilization – USP7 seems to promote POT1 degradation in ALT^+^ cells (Episkopou et al., 2019). Although a precise explanation for this difference remains unclear, it seems to involve the specific localization of ALT^+^ telomeres within promyelocytic leukemia (PML) nuclear bodies. These ALT-associated PML bodies have an essential role in telomere-lengthening in ALT^+^ cells, by bringing chromosome ends together with the recombination proteins that mediate their lengthening (Draskovic et al., 2009; Osterwald et al., 2015). USP7 also associates with PML bodies (Sarkari et al., 2011) and PML proteins are essential for USP7 to degrade POT1 (Episkopou et al., 2019). Here, USP7 is thought to contribute to POT1 degradation by stabilizing one of the many E3 ubiquitin ligases known to accumulate within PML bodies (Cheng and Kao, 2013; Episkopou et al., 2019). Although USP7-regulated degradation of POT1 is likely required for proper ALT regulation, this process is strictly regulated by the USP7 inhibitor protein, TSPYL5 (testis-specific Y-encoded-like protein 5). TSPYL5 competitively binds to the USP7 TRAF-like domain, limiting USP7 substrate-binding (Epping et al., 2011). Regulation of USP7 by TSPYL5 is of particular importance in ALT^+^ cells, as unlike in telomerase^+^ cells, TSPYL5 depletion causes cell death due to unrestricted POT1 degradation (Episkopou et al., 2019).

### p53-Dependent Transcription

Regulating the p53 pathway is one of the most well-studied and well-characterized aspects of USP7 function (Li et al., 2002; Figure 3E). Colloquially referred to as the ‘guardian of the genome’, tumor suppressor protein p53 is an essential transcription factor that stimulates the expression of hundreds of target genes in response to genomic stress. Depending on the type and extent of the damage that occurs, these target genes encode proteins that function in cell cycle arrest, DNA damage repair and apoptosis (Hafner et al., 2019).

In undamaged cells, p53 protein levels are maintained at minimal levels due to prompt proteasome-targeting polyubiquitination by the Mdm2 E3 ubiquitin ligase (Haupt et al., 1997; Honda et al., 1997; Kubbutat et al., 1997). While Mdm2 is also targeted for proteasomal degradation due its auto-polyubiquitination (Fang et al., 2000), USP7 is able to stabilize Mdm2 by rapidly removing these ubiquitin chains (Cummins et al., 2004; Li et al., 2004). Furthermore, USP7 deubiquitinates and stabilizes the Mdm2 interacting partner – and substrate (Kawai et al., 2003; Pan and Chen, 2003) – Mdm4 (also known as Mdmx) (Meulmeester et al., 2005). Mdm4 further contributes to Mdm2 stability by disrupting self-ubiquitination of the latter (Stad et al., 2001), while also stimulating Mdm2-mediated polyubiquitination of p53 (Linares et al., 2003). Further, Mdm4 directly interacts with p53 to disrupts its DNA-binding and transcriptional activation (Francoz et al., 2006; Wei et al., 2016). The interaction between USP7 and Mdm2 is also enhanced by DAXX, which simultaneously binds both proteins and enhances the E3 ubiquitin ligase activity of Mdm2 towards p53 (Tang J. et al., 2006).

The SUV39H1 methyltransferase (also known as KMT1A) is another Mdm2 substrate that is deubiquitinated and stabilized by USP7 in undamaged cells (Bosch-Presegué et al., 2011; Mungamuri et al., 2016). As with Mdm4, SUV39H1 negatively regulates the transcriptional activation of p53. It does so by co-localizing with p53 at the promoters of target genes, where it trimethylates lysine 9 of histone H3 (H3K9me3) to promote formation of transcriptionally inactive higher-order heterochromatin (Mungamuri et al., 2012). SUV39H1 function is also modulated by the SIRT1 deacetylase, which regulates SUV39H1 in two separate ways: (1) by deacetylating SUV39H1 lysine residue 266 (K266) (Vaquero et al., 2007) and (2) by deacetylating lysine 9 of histone H3 (H3K9Ac) to allow SUV39H1 methylation (Vaquero et al., 2004, 2007). As SIRT1 was recently found to be deubiquitinated and stabilized by USP7, these findings suggest another way through which USP7 can regulate SUV39H1 activity and p53 transcription (Song et al., 2020).

The above interactions demonstrate the critical roles USP7 plays in suppressing p53 activation in undamaged cells. USP7 is, however, also important for mounting a strong p53 response following DNA damage. Fundamental to this response is a switch from USP7 deubiquitinating Mdm2 to preferentially deubiquitinating p53 (Li et al., 2004). An obstacle to this switch is that while USP7 binds both Mdm2 and p53 via its TRAF-like domain, Mdm2 makes more extensive contacts and thus binds the TRAF-like domain with a higher affinity (Hu et al., 2006; Sheng et al., 2006). A contributor to this switch is the DNA damage-induced phosphorylation of p53, Mdm2, Mdm4, and DAXX by the Ataxia telangiectasia mutated (ATM) kinase and its downstream effector kinase, Chk2 (Cheng and Chen, 2010; Maréchal and Zou, 2013). These phosphorylation events contribute to disrupting the p53-Mdm2 (Chehab et al., 2000; Shieh et al., 2000), and Mdm2-DAXX (Tang et al., 2013) interactions, as well as to the degradation of Mdm2 and Mdm4 (Stommel and Wahl, 2004; Chen et al., 2005; Pereg et al., 2005, 2006; LeBron et al., 2006). Deacetylation of Mdm2 by Sirt1 also contributes to the Mdm2/p53 switch, by disrupting the association between Mdm2 and USP7, leading to Mdm2 auto-ubiquitination (Nihira et al., 2017).

Various regulatory proteins also influence the transition of USP7 between Mdm2- and p53-binding. The RASSF1A tumor suppressor is one such protein, which disrupts the Mdm2-DAXX-USP7 interaction in response to DNA damage by simultaneously binding to both Mdm2 and DAXX. RASSF1A thereby destabilizes Mdm2, allowing for a p53-dependent delay in G1-S cell cycle progression (Song et al., 2008b). ABRO1 (also known as FAM175B) also contributes to the p53 response by stabilizing the p53-USP7 interaction and promoting USP7-mediated p53 deubiquitination (Zhang J. et al., 2014). The p53-USP7 interaction is also negatively regulated by the TSPYL5 inhibitor protein (Epping et al., 2011). As mentioned in the ‘***Telomere Maintenance***’ section above, TSPYL5 binds to the USP7 TRAF-like domain, competitively inhibiting USP7 substrate binding. TSPYL5 disrupts the p53-USP7 interaction, leading to increased polyubiquitination and degradation of p53. Unlike RASSF1A and ABRO, which stimulates p53-activation, TSPYL5 therefore seems to contribute to a more measured p53 response.

### Nucleotide Excision Repair

Aside from activating p53, multiple studies have suggested that USP7 also has a direct role in the DNA damage response via regulation of the nucleotide excision repair (NER) pathway (Sarasin, 2012; Schwertman et al., 2012; He et al., 2014; Higa et al., 2016; Zhu et al., 2020; Figure 3F). NER is a central pathway through which cells remove a range of bulky DNA lesions, caused by numerous environmental mutagens and metabolic byproducts. While such lesions are structurally diverse and are caused by a range of DNA-damaging agents, UV radiation-induced lesions are a primary substrate (Schärer, 2013). Indeed, cells depleted of USP7 are deficient in the repair of UV-induced cylobutane pyrimidine dimers and 6-4 photoproducts (He et al., 2014).

The detection of DNA lesions and the initiation of NER, occurs through two sub-pathways: global genome NER (GG-NER) and transcription-coupled NER (TC-NER). In GG-NER, lesions are detected throughout the genome by the XPC-Rad23B and DDB1-XPE protein complexes, which recognize helical distortions caused by the DNA adduct. By contrast, TC-NER is activated by the stalling of RNA polymerase II at lesions in the transcribed strand of active genes, as well by accumulation of the TC-NER proteins ERCC8 (CSA), ERCC6 (CSB), and XAB2 at sites of damage. In both GG- and TC-NER, lesion recognition is followed by unwinding of the DNA by TFIIH helicase, excision of the lesion, and the synthesis and ligation of a new DNA patch (Kusakabe et al., 2019).

USP7 has apparent functions in the initiation steps of both GG- and TC-NER pathways. In the former, USP7 has been found to interact with and deubiquitinate the lesion sensing factor, XPC (He et al., 2014). XPC is polyubiquitinated in cells in response to UV-induced DNA damage by a complex containing the DDB1-XPE proteins and the Cul4A E3 ubiquitin ligase (Sugasawa et al., 2005; Wang et al., 2005). Although ubiquitination of XPC is functionally important for enhancing binding to damaged DNA, it does so at the expense of exposing XPC to valosin-containing protein (VCP/p97)-mediated proteolysis. Deubiquitination of XPC by USP7, however, stabilizes XPC and prevents its premature degradation (He et al., 2014).

In the early stages of TC-NER, USP7 interacts with and is recruited to lesions by the UVSSA scaffolding protein. This interaction is important for USP7 to localize and subsequently stabilize the adjacent protein, ERCC6 (Schwertman et al., 2012). Indeed, ERCC6 protein levels are substantially reduced in cells depleted of USP7 or UVSSA (Zhang et al., 2012). Furthermore, while ERCC6 is degraded in cells following UV irradiation (Groisman et al., 2006; Wei et al., 2011), this degradation is more rapid in the absence of either protein. Although ERCC6 degradation is necessary for the resumption of transcription following NER, it has been suggested that USP7 helps to delay this process until the proper completion of ERCC6 function (Schwertman et al., 2012).

### Double-Strand DNA Break Repair

USP7 also has important regulatory roles in the repair of double-strand DNA breaks (DSBs) (Figure 3G). DSBs are an especially toxic form of DNA lesion, as their incorrect repair can lead to chromosomal fragmentation and/or rearrangement (So et al., 2017). Two major DNA repair pathways are employed for the repair of double-strand DNA breaks in cells: non-homologous end-joining (NHEJ) and homologous recombination (HR). NHEJ is a comparatively simple DSB repair pathway, in which either end of a DSB are bound by the Ku70/80 heterodimer, aligned and then re-ligated by DNA ligase IV. If necessary, this process may involve a small degree of end-processing (either gap-filling or ssDNA-trimming) to create compatible DNA ends (Lieber, 2010). By contrast, the initial stages of HR involve the large-scale resection of the 5’-terminating DNA strand to generate 3’-ssDNA tracts of up to 3.5 kb in length (Zhou et al., 2014). Resection of the break is catalyzed by the Mre11-Rad50-Nbs1 (MRN), DNA2, and Exonuclease 1 (Exo1) nucleases, in concert with the BLM helicase (BLM). These tracts are then used to invade a sister chromatid, where they are extended past the break and then reanneal with the second end of the DSB (Brandsma and van Gent, 2012). While NHEJ may be used at any stage during the cell cycle, HR is only available during the S and G2 cell cycle phases, where a sister chromatid is available (Khanna and Jackson, 2001). HR is also the dominant pathway used in the repair of “single-ended DSBs” caused by the collapse of replication forks (Whelan et al., 2018).

The depletion of USP7 from cells has been found to greatly compromise the repair of DSBs by either NHEJ or HR (Wang et al., 2016; An et al., 2017). This is due to a key role of USP7 in the early recruitment of DNA repair proteins to sites of DSB lesions. One such protein is the histone demethyltransferase, PHF8. As discussed in our “***Cell Cycle Regulation***” section above, PHF8 is an interactor of USP7, which the latter stabilizes via deubiquitination. In addition to the transcriptional regulation of cyclin A2, PHF8 interacts with Ku70 and the BLM helicase to facilitate their localization to sites of DSBs. Indeed, the recruitment of either protein is impaired in cells following PHF8 or USP7 depletion (Wang et al., 2016). As Ku70 and BLM are essential factors in NHEJ and HR, respectively, USP7 is thus able to influence both pathways.

USP7 also regulates the protein stability of mediator of DNA damage checkpoint 1 (MDC1) (Su et al., 2018). MDC1 is a binding partner of the MRN nuclease complex (Goldberg et al., 2003) and functions in the initial detection and signaling of DSBs (Stewart et al., 2003). This is demonstrated by the failed recruitment of the downstream DNA repair proteins 53BP1 and BRCA1 in MDC1-depleted cells following DNA damage; this defect is mimicked in cells depleted of USP7 (Su et al., 2018). MDC1 promotes DSB signaling by binding to histone H2AX following its phosphorylation by the repair kinase, ataxia-telangiectasia mutated (ATM) (Stewart et al., 2003). In addition, MDC1 is itself phosphorylated by ATM, which creates a binding site for the RNF8 E3 ubiquitin ligase (Kolas et al., 2007; Mailand et al., 2007). The accumulation of RNF8 subsequently triggers the recruitment of another E3 ubiquitin ligase, RNF168 (Nowsheen et al., 2018), which catalyzes the K63-linked polyubiquitination of histones H2A and H2AX (Mattiroli et al., 2012). These ubiquitin chains are in-turn bound by the ubiquitin-binding domains of 53BP1, and the BRCA1-interacting protein, Rap80 (Sobhian et al., 2007; Fradet-Turcotte et al., 2013).

In addition to stabilizing MDC1 – and thus promoting RNF168 accumulation – USP7 also directly deubiquitinates and stabilizes RNF168 (Zhu et al., 2015), as well as its paralog, RNF169 (An et al., 2017). While RNF168 promotes 53BP1 and BCRA1 localization to sites of DSBs, RNF169 is a direct inhibitor of this binding. This is because, once RNF168 synthesizes H2A/H2AX ubiquitin chains, RNF169 competes with 53BP1 and BRCA1 for ubiquitin binding (Chen et al., 2012; Panier et al., 2012; Poulsen et al., 2012). Although it seems counterintuitive for USP7 to both promote and limit 53BP1 and BRCA1 accumulation at sites of DSBs, an explanation may come from the finding that while 53BP1 and BRCA1 promote DSB signaling, 53BP1 subsequently prevent DSB resection during HR (Bunting et al., 2010; Coleman and Greenberg, 2011). USP7 might therefore have an important role in balancing these activities, to allow for both HR activation as well as subsequent DSB resection.

USP7 also influences DSB repair by regulating checkpoint kinase 1 (Chk1) (Faustrup et al., 2009; Alonso-de Vega et al., 2014; Zhang P. et al., 2014). Chk1 is activated during HR following the resection of DSBs and the exposure of single-stranded DNA. This elicits activation of the ATR kinase, which stimulates Chk1 activity via phosphorylation (Choi et al., 2007). Chk1 then phosphorylates numerous downstream targets, including the Cdc25A phosphatase (Uto et al., 2004), which prompts Cdc25A proteasomal degradation (Mailand et al., 2000; Xiao et al., 2003). Cdc25A is a dual-specific phosphatase, which promotes cell cycle progression by removing inhibitory phosphate modifications from cyclin dependent kinases. The degradation of Cdc25A during HR thus prevents cell cycle progression, triggering S and G2 cell cycle arrest (Donzelli and Draetta, 2003).

USP7 controls Chk1 signaling by deubiquitinating and stabilizing both Chk1 and its activator protein, Claspin (Faustrup et al., 2009; Alonso-de Vega et al., 2014; Zhang P. et al., 2014). Here, the interaction between USP7 and Chk1 is enhanced by ZEB1, which is itself phosphorylated and stabilized by the ATM kinase, in response to DNA damage (Zhang P. et al., 2014). Interestingly, USP7 also negatively regulates Chk1-mediated cell cycle arrest, by deubiquitinating and stabilizing Cdc25A in response to DNA damage. Here, USP7 is recruited via an interaction with the Cdc25A-interactor, BRE (Biswas et al., 2018). Although the precise purpose of this opposing regulation is not fully understood, one explanation might be that by maintaining a small pool of Cdc25A, USP7 might play a role in cell cycle re-entry, following DSB repair. Such a model will, however, require further investigation.

### DNA Damage Bypass

Another way USP7 regulates the cellular response to DNA damage is via the specialized replication pathways of DNA damage bypass (Zlatanou and Stewart, 2015; Figure 3H). Unlike the NER and DSB repair pathways, DNA damage bypass does not mediate the repair of damaged DNA, but rather allows the cell to avoid DNA lesions encountered during replication, so they may be repaired at a later time (Sale, 2012). These lesions include bulky DNA adducts such as those repaired by NER.

The synthesis of DNA during normal replication is facilitated by the three “replicative” DNA polymerases: Pol α, Pol δ and Pol ε (Johansson and Dixon, 2013). In this process, high accuracy is essential to prevent the inadvertent accumulation of DNA mutations. A major contributor to this accuracy is the intrinsic high-fidelity of the replicative polymerases (Bębenek and Ziuzia-Graczyk, 2018). This is largely ensured by the tight active site of these polymerases, which geometrically accommodates the template DNA and only those dNTPs that can form a correct base pair (Beard and Wilson, 2003). However, while the active site of the replicative polymerase is suited to replicating undamaged DNA, it is unaccommodating if the polymerase encounters a DNA lesion on the template strand. In such cases, DNA damage becomes a replication barrier, which must be overcome to ensure the DNA is completely duplicated prior to cell division. Furthermore, in the absence of a timely restart of replication, stalled replication forks can collapse to generate toxic DSBs, which pose further risk to the cell (Saintigny et al., 2001).

To overcome replication lesions, the cell employs one of two pathways of DNA damage bypass: translesion synthesis (TLS) and template switching (Sale, 2012). In the former, replicative polymerases that have stalled due to a bulky DNA lesion are replaced by specialized TLS polymerases, which are able to bypass the damaged residues (Sale et al., 2012). Such bypass is facilitated by the comparatively more open active sites of TLS polymerases, which can accommodate alterations to the template strand (Yang, 2003). TLS, however, comes at the expense of potential misincorporation of incorrect dNTPs, due to the intrinsic lower fidelity of the translesion polymerase active sites (Beard et al., 2002). By contrast, template switching is considered a more accurate form of DNA damage tolerance. Here, the stalled replicative polymerase makes use of the undamaged, newly synthesized strand on the sister chromatid. This reaction occurs via a strand invasion mechanism, similar to that employed during HR (Giannattasio et al., 2014).

Recent reports have suggested that USP7 has a role in the initial stages of both translesion synthesis and template switching. Indeed, following UV exposure, cells depleted of USP7 are defective at elongating nascent DNA strands (Zlatanou et al., 2016). This defect seems due to a role of USP7 in regulating ubiquitination of the PCNA sliding clamp (Kashiwaba et al., 2015; Qian et al., 2015; Masuda et al., 2019). During translesion synthesis, the PCNA sliding clamp is monoubiquitinated at lysine residue 164 (K164) by the E3 ubiquitin ligase, Rad18 (Watanabe et al., 2004). This modification stimulates recruitment of translesion synthesis polymerases, which bind the PCNA ubiquitin group via their ubiquitin-binding domains (Kannouche et al., 2004; Bienko et al., 2005). In template switching, the monoubiquitin moiety added to K164 of PCNA is instead further ubiquitinated by the HLTF E3 ubiquitin ligase, to form a K63-linked ubiquitin chain (Motegi et al., 2008; Unk et al., 2008). Recently, K63-linked polyubiquitination of PCNA was found to stimulate template switching by creating a binding platform for the DNA translocase, ZRANB3 (Vujanovic et al., 2017).

Research over the past decade has suggested an intricate manner through which USP7 fine-tunes PCNA ubiquitination. Most directly, USP7 has been reported to deubiquitinate PCNA during interphase of the cell cycle, to suppress PCNA monoubiquitination in response to oxidative – and to a lesser extent, UV-induced – DNA damage (Kashiwaba et al., 2015). Although it is yet to be demonstrated in cells, biochemical assays have suggested USP7 might also be able to deubiquitinate PCNA modified by the addition of K63-linked ubiquitin chains (Masuda et al., 2019). These observations suggest that USP7 might deubiquitinate PCNA to negatively regulate both the translesion synthesis and template switching pathways.

In addition, USP7 has been suggested to indirectly influence PCNA mono-ubiqutination, by regulating the stability of the Rad18 and HLTF E3 ubiquitin ligases (Qing et al., 2011; Zlatanou et al., 2016). Indeed, depleting USP7 from cells was found to decrease the protein levels of Rad18 and HLTF, suppress PCNA mono and polyubiquitination, as well as reduce Pol η foci formation in UV irradiated cells. These findings are, however, complicated by the finding that, in addition to stabilizing HLTF, USP7 also deubiquitinates the HLTF E3 ubiquitin ligase, CHFR (Oh et al., 2007). USP7 may therefore act to balance the level of HLTF in the cell, by promoting both polyubiquitination (via CHFR) and deubiquitination of the protein. These reports further demonstrate the intricacies through which USP7 influences PCNA ubiquitination.

USP7 has also been proposed to regulate the stability of the TLS polymerase, DNA polymerase eta (Pol η) (Qian et al., 2015). Here, USP7 seems to regulate Pol η transcriptionally – via p53 (Lerner et al., 2017) – as well as through protein stabilization by directly removing K48-linked polyubiquitin chains. Furthermore, as Pol η is polyubiquitinated by Mdm2 (Jung et al., 2012), which is itself a substrate of USP7 (Li et al., 2004), these observations suggest that USP7 regulates Pol η protein levels both positively (by direct deubiquitination) and negatively (by stabilizing Mdm2). Indeed, as evidence of this complexity, both the depletion and over-expression of USP7 in cells were reported to enhance Pol η protein levels (Qian et al., 2015). It should be noted, however, that despite the attractive elegance of this proposed model, these claims have been contradicted by a subsequent publication, where Pol η protein levels appeared unchanged following siRNA-mediated depletion of USP7 (Zlatanou et al., 2016). Further work will, therefore, be required to unravel these apparent inconsistencies.

### Apoptosis

The final genome stability pathway we will discuss is that of apoptosis (Figure 3I). Unlike the pathways above, which are important for preventing cells from developing DNA mutations, apoptosis is essential for removing cells which have suffered irreparable genomic damage (Zhivotovsky and Kroemer, 2004; Borges et al., 2008). Apoptosis is thereby essential to prevent the propagation of cells whose genomic integrity has been compromised (Zhivotovsky and Kroemer, 2004).

As discussed in previous sections, USP7 has a central role in stabilizing p53 protein levels in response to genomic stress (Sheng et al., 2006). This allows p53 to participate in the DNA damage response, by transcriptionally activating genes involved in cell cycle arrest and DNA damage repair. Beyond a certain level of DNA damage, however, p53 switches to a pro-apoptotic function, where it transcriptionally activates a distinct set of gene targets (Fridman and Lowe, 2003). This includes pro-apoptotic members of the Bcl-2 family (e.g., PUMA, Bax), which mediate mitochondrial outer membrane permeabilization and subsequent caspase activation, to dismantle the cell (Nakano and Vousden, 2001; Chipuk et al., 2004; Aubrey et al., 2018).

The switch between cell cycle arrest and apoptosis in response to DNA damage, is largely dictated by the cell reaching threshold p53 protein levels (Kracikova et al., 2013; Paek et al., 2016). This is driven by aspects of USP7 function we have considered in previous sections. In addition, however, the apoptotic function of p53 is activated by a series of post-translational modifications. This includes acetylation of lysine residue 120 (K120) by the Kat5 histone acetyltransferase (also known as Tip60). K120 resides within the p53 DNA-binding domain and its acetylation increases its binding affinity for the *Bax* and *PUMA* gene promoters, while simultaneously preventing binding to the promoter of p21 (Sykes et al., 2006; Tang Y. et al., 2006; Reed and Quelle, 2014). In addition to stabilizing p53 protein levels, USP7 contributes to p53-dependent apoptosis through interacting with and de-ubiquitinating Kat5. Further, USP7 was found to promote p53 K120 acetylation, as well as *PUMA* expression and apoptosis following DNA damage (Dar et al., 2013).

## USP7: Links With Cancer

In the above sections we have discussed the numerous and intricate manners through which USP7 regulates genome stability. In the following section, we will now discuss links between USP7 deregulation and carcinogenesis, as well as strategies for targeting USP7 in anti-cancer therapy.

### USP7 Has Context-Dependent Tumor Suppressor and Oncogenic Roles in Cancer Progression

USP7 was initially described as a having a tumor suppressive role, following its identification as a p53 deubiquitinating enzyme (Li et al., 2002; Bhattacharya et al., 2018). Consistent with this assessment, USP7 gene expression has been found to be frequently downregulated in non-small cell lung adenocarcinomas, where low USP7 mRNA expression correlated with reduced p53 immunostaining (Masuya et al., 2006). These clinical findings reflect observations in cell lines, where partially – although not completely – depleting USP7 causes a reduction in p53 protein levels (Li et al., 2004). Furthermore, low USP7 expression levels were found to be an indicator of poor patient prognosis, supporting a role for USP7 in suppressing cancer progression by maintaining genome stability (Masuya et al., 2006).

While the tumor suppressive role of USP7 has been largely attributed to regulating p53, USP7 evidently also prevents genetic alterations through an assortment of p53-independent means, given its intricate roles in maintaining genomic stability. For instance, USP7 mRNA expression levels were found to correlate with genomic instability across the NCI-60 panel of cancer cell lines (Giovinazzi et al., 2014). Here, genomic instability was detected based on karyotypic complexity (Roschke et al., 2003). Although p53 status was not a reported parameter in these analyses, similar chromosomal abnormalities were detected in both p53 positive and null cell lines following USP7 depletion, suggesting this phenotype might be caused by means other than p53 deregulation (Giovinazzi et al., 2013, 2014). A complete understanding of how each individual USP7 function contributes to tumor suppression, however, remains unclear and will require further investigation.

In addition to tumor suppressive roles, USP7 also seems to have context-dependent oncogenic functions (Bhattacharya et al., 2018). Indeed, in one study, a non-monotonic relationship was observed between USP7 expression and breast cancer survival, where both low and high levels of USP7 were associated with poor outcome (Hernández-Pérez et al., 2017). In this study, USP7 and Geminin protein levels were strongly correlated in a cohort of invasive breast cancers. High Geminin protein levels are prognostic of poor clinical outcome and are associated with genome instability, DNA replication errors and aneuploidy (Sundara Rajan et al., 2014). As Geminin is a substrate of USP7, these findings suggest USP7 over-expression contributes to breast cancer progression by stabilizing Geminin (Hernández-Pérez et al., 2017).

A strong correlation has also been observed between USP7 and the cell cycle regulatory protein PHF8 (Wang et al., 2016). This correlation was observed in clinical breast cancer samples and increased with histological grade. As we discussed in the ***Cell Cycle Regulation*** section, USP7 deubiquitinates and stabilizes PHF8, allowing the latter to upregulate gene targets involved in cell cycle progression, including cyclin A2 (Liu et al., 2010; Wang et al., 2016). Indeed, USP7, PHF8 and cyclin A2 were all found to be upregulated in several breast cancers, as well as in colon and rectum cancers, compared to adjacent tissues (Wang et al., 2016). As cyclin A2 deregulation is associated with aberrant cell proliferation (Gopinathan et al., 2014), these findings suggest another way in which USP7 upregulation may promote oncogenesis.

USP7 might also contribute to oncogenesis by deubiquitinating and stabilizing Ki-67 (Zhang et al., 2016). As with Cyclin A2, Ki-67 is highly expressed in malignant tissues and promotes cell division (Sales Gil and Vagnarelli, 2018; Horie et al., 2019). This interaction may be particular disease-relevant in non-small cell lung cancer cells, where a strong positive correlation between the two proteins has been observed (Zhang et al., 2016).

Although outside the scope of this review, USP7 likely also contributes to carcinogenesis by regulating a number of tumor suppressors and oncogenes with roles outside of direct genome stability maintenance (Bhattacharya et al., 2018). These include the tumor suppressors PTEN (Song et al., 2008a), NOTCH1 (Shan et al., 2018), the Foxo proteins (van der Horst et al., 2006) and the retinoblastoma protein (Bhattacharya and Ghosh, 2014), as well the oncoproteins c-Myc (Bhattacharya and Ghosh, 2015), the REST transcription factor (Huang et al., 2011) and β-catenin (Novellasdemunt et al., 2017). Together, these data demonstrate that USP7 has context-dependent tumor suppressor and oncogenic roles and that up- or down-regulation can contribute to carcinogenesis.

### Targeting USP7 in Cancer Therapy

**FIGURE 4:**
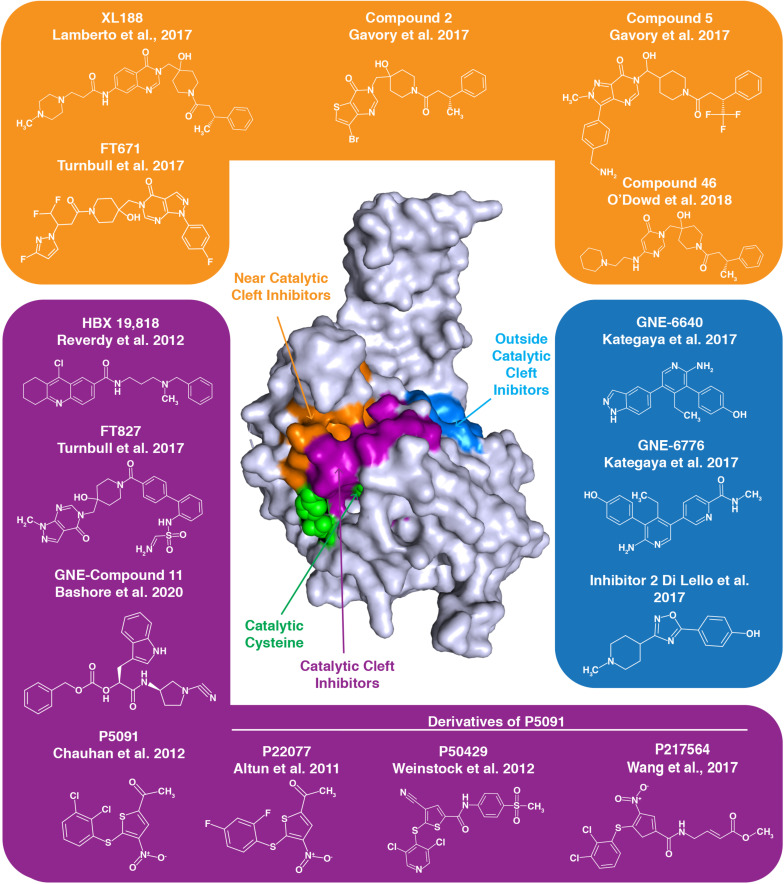
Numerous inhibitors of USP7 have been developed. The structure represents the USP7 catalytic domain. Green spheres on the USP7 catalytic domain represent catalytic triad active site residues C223, H464, and D481. USP7 inhibitors indicated with a purple background target the catalytic cleft, those with an orange background bind near the catalytic cleft and those with a blue background bind outside the catalytic cleft. The binding region for each group of inhibitors is demonstrated by the correspondingly colored surfaces of the USP7 catalytic domain.

The frequent up-regulation of USP7 in many cancer types and the apparent contributions of this upregulation to carcinogenesis, has led to speculation that USP7 could be an effective target in anti-cancer therapies. Enthusiasm for this approach is demonstrated by the dozens of small molecule USP7 inhibitors developed in the past few years. While human trials of USP7 inhibitors have yet to be conducted, many of these compounds have been found to inhibit cancer cell growth *in vitro*, as well as in animal xenograft models (Fan et al., 2013; Agathanggelou et al., 2017; Turnbull et al., 2017; Peng et al., 2019; Zhang et al., 2020). The molecular basis of the interaction between several inhibitors and USP7 has been determined, revealing that these small molecules largely target the USP7 catalytic domain [Figure 4 and Table 2; for a recent and extensive review, see Li and Liu (2020)].

**TABLE 2:** Available structures and strength of interactions of select USP7 inhibitors.

**USP7 inhibitor**	**Strength of interaction with USP7 catalytic domain**	**PDB ID**
XL188	K_d_ = 90 nM	5vs6
Compound 46	IC_50_ = 0.09 μM	6f5h
Compound 2	IC_50_ = 0.3 μM	5n9r
Compound 5	IC_50_ = 22.0 nM	5n9t
FT671	K_d_ = 65 nM	5nge
FT827	K_d_ = 7.8 μM	5ngf
5091	IC_50_ 4.2 μM	N/A
217564	IC_50_ = 3 μM	N/A
GNE-6776	IC_50_ = 0.61 ± 0.15 μM	5uqx
GNE-6640	IC_50_ = 0.43 ± 0.07 μM	5uqv
Inhibitor 2	IC_50_ = 79 μM	5whc
GNE Compound 11	IC_50 =_ 4.2 μM	N/A

The quinazolin-based compound XL188 is one such inhibitor that was developed by a structure-guided approach. XL188 non-covalently binds the USP7 catalytic domain near the active site cleft. The inhibitor in complex with the USP7 catalytic domain reveals that binding occurs about 5 Å from the catalytic triad (Lamberto et al., 2017). Similarly, the non-covalent pyrimidine-based USP7 inhibitor Compound 46 binds a few angstroms from the active site residues (O’Dowd et al., 2018).

High-throughput screening led to the development of non-covalent pyrimidone-based inhibitors, including Compounds 2 and 5. These compounds both bind over 5 Å away from C223 (Gavory et al., 2018).

Interestingly, USP7 pyrimidine-based FT class inhibitors target different sites of the USP7 catalytic domain. The non-covalent inhibitor FT671 in complex with the catalytic domain reveals this inhibitor is several angstroms from C223, similar to XL188. Inhibitor FT827 in complex with the catalytic domain reveals that this inhibitor enters the active site cleft and covalently modifies C223. This covalent modification of C223 prevents it from acting as a nucleophile and hydrolyzing isopeptide bonds (Turnbull et al., 2017). Despite differences in binding, both USP7-FT inhibitor complexes reveal a misaligned catalytic triad and the switching loop being in an unproductive conformation.

Like the covalent FT827 inhibitor, several other USP7 inhibitors also covalently modify the catalytic cysteine to inactivate USP7. USP7 nitrothiophene-based inhibitor 5091 proved to be a promising therapeutic for treatment of multiple myeloma (Chauhan et al., 2012), and its second generation inhibitor, 217564, was determined to covalently modify C223 to inhibit USP7 activity (Wang et al., 2017). P22077 and P50429, also derivatives of 5091, both irreversibly modify C223. Here, mutation of C223 to an alanine prevents inhibitor binding to USP7, revealing specificity for the catalytic cysteine (Pozhidaeva et al., 2017). While the USP7 IC_50_ for P50429 and P22077 are in the low micromolar range, these inhibitors are not specific to USP7 as they also target the USP7 homolog USP47 (Tian et al., 2011; Weinstock et al., 2012). USP7 inhibitor HBX 19,818 also specifically and covalently targets C223 with low micromolar affinity (Reverdy et al., 2012).

Interestingly, some USP7 inhibitors work in an allosteric manner, by binding distally to the active site to inhibit USP7 activity. USP7 catalytic domain in complex with aminopyridine-based inhibitors GNE-6776 and GNE-6640 reveals these interactions occur 12 Å from the active site cleft. Rather than interfering with the active site residues, this class of inhibitors disrupts USP7-ubiquitin binding. With IC_50_ values for the catalytic domain in the low micromolar range, these GNE class inhibitors are unfortunately not specific to USP7, as other USPs are inhibited by these small molecules (Kategaya et al., 2017). USP7 Inhibitor 2, which was part of the GNE-inhibitor screen, also binds distal to the catalytic site (Di Lello et al., 2017).

Recently, a new class of GNE cyanopyrrolidine-based inhibitors has been described. These inhibitors, including GNE Compound 11, specifically target C223. Interestingly, these novel inhibitors cause desulfhydration of the active-site cysteine of USP7, converting it to dehydroalanine (DHA). Therefore, the ability of C223 to act as a nucleophile for bond hydrolysis is eliminated (Bashore et al., 2020).

USP7 inhibitors predominantly kill cancer cells by inducing p53-dependent apoptosis (Schauer et al., 2020). One way through which this occurs is via the stabilization of p53, resulting from the degradation of Mdm2 in the absence of its USP7-mediated deubiquitination (Li et al., 2002, 2004; Cummins et al., 2004; Becker et al., 2008; Kategaya et al., 2017). In addition, USP7 inhibitors simultaneously promote p53 activation via the induction of genomic instability (Giovinazzi et al., 2013, 2014; Peng et al., 2019). For instance, as we discussed in the ***Mitosis*** section above, USP7 inhibition causes mitotic stress as a result of Plk1 depletion, driving cells towards apoptotic cell-death (Peng et al., 2019). Interestingly, while some studies have reported a strict dependency on p53 for USP7 inhibitor-induced cell death (Fan et al., 2013; Schauer et al., 2020), others have suggested the inhibition of USP7 may cause cell death even in the absence of functional p53, possibly due to deregulation of other essential cellular pathways (Chauhan et al., 2012; Dar et al., 2013; Kategaya et al., 2017).

Aside from monotherapy-type approaches, several publications in the past decade have indicated USP7 inhibitors might also be effective in cancer treatment, in combination with other chemotherapeutic agents. For instance, P22077 has been found to significantly enhance the efficacy of the DNA damaging compounds doxorubicin and etoposide to kill cultured neuroblastoma cells (Fan et al., 2013). While sensitization by P22077 was, in this study, found to be dependent on a functional USP7-HDM2-p53 axis, others have found USP7 inhibition may sensitize cancer cells to DNA damaging agents, even in p53 defective tumors. USP7 inhibition with the compound HBX19, 818, for example, was shown to sensitize chronic lymphoblastic leukemia cells to the chemotherapeutic agents cyclophosphamide and mitomycin C in cells with defects in p53 or the ATM kinase (Agathanggelou et al., 2017; Sampath, 2017). Such co-treatment may be particularly effective in cancers that overexpress USP7. For instance, USP7 has been found to be upregulated in many cervical carcinomas, where its expression positively correlates with that of MDC1, as well as with histological tumor grade. Inhibition of USP7 with the compounds GNE-6640 or GNE-6776, however, destabilizes MDC1, as well as sensitizes cervical cancer cells to ionizing radiation-induced genotoxic insult (Su et al., 2018). USP7 has also been suggested as a potential target for sensitization in breast cancer treatment, where USP7 is frequently upregulated and confers resistance to genotoxic insult, by stabilizing PHF8 (Wang et al., 2016).

Another proposed use for USP7 inhibitors is in sensitizing taxane-resistant tumors to taxane therapies (Giovinazzi et al., 2013; Zhang et al., 2016; Peng et al., 2019). Taxanes, such as paclitaxel and docetaxel, kill cancer cells by binding to and stabilizing β-tubulin on the inner surface of microtubules, increasing their polymerization (Jordan et al., 1993). This binding prevents proper mitotic cell division, as although chromosomes can be attached to taxane-stabilized microtubules, the disruption to microtubule dynamics precludes proper tension being established across sister chromatids (Kelling et al., 2003). Taxanes, in this way, prevent proper chromosome orientation on the metaphase plate, leading to activation of the spindle-assembly checkpoint and stalling cells at the metaphase to anaphase transition (Musacchio and Salmon, 2007; Gascoigne and Taylor, 2009). Prolonged stalling at this boundary leads to eventual cell death (Rieder and Maiato, 2004). While paclitaxel and docetaxel are front-line chemotherapeutics for treating breast, prostate and nasopharyngeal carcinomas, resistance to these compounds often develops (Murray et al., 2012). USP7 may contribute to such resistance via its roles in stabilizing PLK1. Indeed, USP7 and PLK1 were found to correlate in tissue sections of primary breast cancer and to be highly expressed in taxane-resistant tumors (Peng et al., 2019). Here, PLK1 over-expression likely contributes to taxane resistance by regulating microtuble dynamics and microtubule-kinetochore attachment (Gutteridge et al., 2016). By inducing PLK1 degradation, USP7 inhibition with P5091 was, however, found to sensitize taxane-resistant cancer cells to paclitaxel and docetaxel and to trigger apoptotic cell death (Peng et al., 2019). These findings suggest another prospective use for USP7 inhibitors in cancer treatment.

## Conclusion

In this review article, we have discussed the extensive roles of USP7 in maintaining the integrity of the genome. Improper activity of USP7 leads to destabilization of its many substrates, resulting in genetic alterations that may drive tumorigenesis. Recently, USP7 has become an attractive pharmacological target for inducing apoptotic cell death in cancer cells. This appreciation has skyrocketed the development of small-molecule compounds targeting USP7. With relentless high-throughput screens and optimizations being pursued, human trials of USP7 inhibitors may be on the horizon.

## Author Contributions

GV and NA wrote the first draft of the manuscript, prepared the figures, and established the scope of the review. IB and RW assisted with structuring the manuscript and editing the final text. All authors read and approved the final version prior to submission.

## Conflict of Interest

The authors declare that the research was conducted in the absence of any commercial or financial relationships that could be construed as a potential conflict of interest.
